# Using Hospital Discharge Database to Characterize Chagas Disease Evolution in Spain: There Is a Need for a Systematic Approach towards Disease Detection and Control

**DOI:** 10.1371/journal.pntd.0003710

**Published:** 2015-04-17

**Authors:** Zaida Herrador, Eva Rivas, Alin Gherasim, Diana Gomez-Barroso, Jezabel García, Agustín Benito, Pilar Aparicio

**Affiliations:** 1 National Centre for Tropical Medicine, Health Institute Carlos III (ISCIII in Spanish), Madrid, Spain; 2 Network Biomedical Research on Tropical Diseases (RICET in Spanish), Madrid, Spain; 3 Department of Preventive Medicine, University Hospital Nuestra Señora de la Candelaria, Tenerife, Spain; 4 Biomedical Research Centre Network for Epidemiology and Public Health (CIBERESP), Health Institute Carlos III (ISCIII in Spanish), Madrid, Spain; Institut de Recherche pour le Développement, BENIN

## Abstract

After the United States, Spain comes second in the list of countries receiving migrants from Latin America, and, therefore, it is the European country with the highest expected number of infected patients of Chagas disease. We have studied the National Health System’s Hospital Discharge Records Database (CMBD) in order to describe the disease evolution from 1997 to 2011 in Spain. We performed a retrospective descriptive study using CMBD information on hospitalizations including Chagas disease. Data was divided in two periods with similar length in time: 1997-2004 and 2005-2011. Hospitalization rates were calculated and clinical characteristics were described. We used multivariable logistic regression to calculate adjusted odds-ratio (aOR) for the association between various conditions and being hospitalized with organ affectation. A total of 1729 hospitalization records were identified. Hospitalization rates for the two periods were 18 and 242.8/100000 population, respectively. The median age was 35 years (range 0-87), 74% were female and the 16-45 age-group was mostly represented (69.8%). Overall, 23.4% hospitalizations included the diagnosis of Chagas disease with organ complications. Being male [aOR: 1.3 (1.00-1.77)], aged 45 and 64 years [aOR: 2.59 (1.42-4.71)], and a median hospitalization cost above 3,065 euro [aOR: 2.03 (3.73-7.86)] were associated with hospitalizations with organ affectation. Since 2005, the number of detected infections increased in Spain. The predominant patients’ profile (asymptomatic women at fertile age) and the conditions associated with organ affectation underlines the need for increased efforts towards the early detection of *T cruzi*.

## Author Summary

Chagas disease, caused by the protozoa *Trypanosoma cruzi*, is endemic in most Latin American countries and it is considered a neglected tropical disease (NTD). *T*. *cruzi* transmission is feasible in vector-free world regions. The main non-vectorial routes are congenital transmission, blood transfusion, and solid organ transplant. According to WHO, in Spain, which is the second country in the world receiving migrant population from endemic countries, there are up to 40.000 persons at risk of developing symptomatic Chagas disease. Since 2005, serological screening for at-risk blood and solid organ donors is mandatory. Lately, some regulations regarding screening of pregnant women from Latin American endemic countries have also been implemented, but only in some autonomous regions. Up to date, there is no surveillance system in place. Thus, the Chagas disease burden remains unknown. In this paper we use hospitalized Chagas disease discharge data, in order to describe Chagas disease’s geographical and temporal trends before and after the implementation of control measures in Spain from 1997 to 2011. We also assess patients’ main clinical characteristics by organ affectation.

## Introduction

Chagas disease, also known as American trypanosomiasis, is a potentially life-threatening illness caused by the protozoan parasite, *Trypanosoma cruzi* (*T*. *cruzi*). This disease presents itself in two phases. The initial, acute phase lasts for about two months after infection. In most cases, symptoms are absent or mild, but can include fever, headache, enlarged lymph glands, pallor, muscle pain, difficulty in breathing, swelling and abdominal or chest pain [1,2]. During the chronic phase, the parasites are hidden mainly in the heart and digestive muscle. Up to 30% of patients suffer from cardiac disorders (sudden death, complex arrhythmias, ventricular aneurysms, heart failure, and thromboembolism) and up to 10% suffer from digestive (typically enlargement of the esophagus or colon), neurological or mixed alterations [3]. In later years the infection can lead to sudden death or heart failure caused by progressive destruction of the heart muscle [1]. Chagas disease can be effectively treated with benznidazole and also nifurtimox. Both drugs are almost 100% effective in curing the disease if given soon after infection at the onset of the acute phase [4]. However, the efficacy of both diminishes the longer a person has been infected, thus the importance of an early diagnosis [5]. Additionally, specific treatment for cardiac or digestive manifestations may be required at latest stages of the infection [6].

Worldwide, between seven and eight million people are estimated to be infected with Chagas. The disease is endemic in 21 Latin American countries [7], where it is mostly transmitted to humans through the bite of the triatomine bug, Chagas disease natural reservoir [8]. Nevertheless, *T*. *cruzi* transmission is also possible in vector-free world regions [9]. The main non-vectorial routes are congenital transmission, blood transfusion, and solid organ transplant, these routes being characteristics but not exclusive to non-endemic countries [10].

Although most of the cases occurr in Latin America [11], Chagas disease in non-endemic countries, such as North America and the Western Pacific Region (mainly Australia and Japan), has come to light since the beginning of 2000, and only more recently in Europe [12]. In 2010, the World Health Organization (WHO) estimated that 80.000 persons could be infected in Europe, making Chagas disease one of the predominant emerging parasitic infections in the Old World [13].

The emergence of the disease in non-endemic countries is mainly linked to population mobility, notably migration from endemic countries [12]. Beginning with 2010, Spain had the highest immigration rates from Latin America within the EU, (second after the USA worldwide) [14]. A study published by the European Center for Disease Prevention and Control (ECDC) estimated that in 2009, 53% (more than 1.7 million people) of the European migrant population coming from Latin America were living in Spain [15]. The same study indicated Spain as the country with the highest prevalence of Chagas disease in migrant population from endemic countries (2.3–3.8%). Estimates that take into account the prevalence of *T*. *cruzi* infection in Latin America suggest that between 40,000 and 65,000 infected people currently reside in Spain [16].

In the last decade some progress has been made regarding the management of Chagas disease in Spain. Since September 2005, in accordance with the Royal Decree RD1088/2005, serological screening of the population considered to be at risk is mandatory in all blood transfusion centers [17]. Regulations regarding screening of pregnant women from Latin American endemic countries and their newborns are in place in 4 out of 17 autonomous regions (Valencia, Catalonia, Galicia and Basque Country) [18–21]. In the rest of the country, the detection of congenital Chagas cases depends mainly on the initiative of the health professionals of the National Health System [15] (Table 1).

**Table 1:** Autonomous regions with local screening programs for the detection of *T*. *cruzi* infection, Spain.

Autonomous region	Publication year	Overall región coverage	Targeted population		
			Pregnant Latin American women and their newborns	Pregnant Latin American women’s relatives	Other categories
Catalonia	2010	Yes	*		
Galicia	2012	Yes	*		
Madrid^1^	2008	No	*	*	
Murcia^2^	2006	No			*
	2013	Yes			*
Basque Country ^3^	2008	Yes	*		*
Valencian Community	2007	Yes	*		

^1^ Hospitals network screening protocol: testing of the other children of an infected mother.

^2^ Immigrant children health care protocol: testing of Bolivian children with positive family history of Chagas disease.

^3^ Recommendations for migrant adult healthcare: Testing of migrants from endemic countries in case of Chagas disease symptomatology, HIV infection, pregnancy with Chagas symptoms (clinical or EKG).

Up to date, there is no surveillance system for Chagas disease implemented in Spain. However, hospitalized cases are recorded within the National Health System′s Hospital Discharge Records Database (CMBD in Spanish) belonging to the Spanish Ministry of Health. In this paper, we describe for the first time the Chagas disease related hospitalizations in Spain between 1997 and 2011, in terms of time, geographical distribution, and disease related individual characteristics.

## Methods

We performed a retrospective descriptive study using CMBD information on Chagas disease related hospitalizations in Spain between January 1^st^, 1997 and December 31^st^, 2011. CMBD database receives notification from around 98% of the public hospitals in Spain [22]. Compulsory health insurance covers an estimated 99.5% of the Spanish population, although persons not covered by health insurance can receive treatment in public hospitals. Since 2005, CMBD also has a gradual coverage from private hospitals [23].

All CMBD’s hospital discharges having included the Chagas disease diagnosis were reviewed, in any of 14 possible diagnostic positions. For each entry, we collected socio-demographic (sex, age and autonomous region of residency) and clinical data (type and department of admission, average length of hospitalization, non-invasive procedures and history of surgical intervention during the hospitalization, re-admission, outcome, hospitalization′s cost to the health care system, financing regime and diagnosis related group (DRG). Other concurrent clinical diagnoses, in any of the 14 positions, were also assessed. International Classification of Diseases, Ninth Revision, Clinical Modification (ICD 9 CM) were used for this purpose [24].

We calculated the hospitalization rates by sex and autonomous regions using as denominators the Latin American officially registered population in Spain from the 21 countries endemic for Chagas disease. Latin American population figures were available since 1998 from the Spanish National Institute of Statistics (INE) website [25]. The rates increase among the two study periods (1998–2004 and 2005–2011) were mapped using the Geographical Information System Arcgis version 10.0.

We described the clinical characteristics separately for: a) all hospitalizations, regardless the Chagas diagnosis position; b) hospitalizations with Chagas as first diagnosis and; c) hospitalization with child delivery related stay as first diagnosis. For all hospitalizations, we also examined the differences between the patients notified with Chagas disease with and without an organ (heart or digestive system) complication. We assessed the group distribution by using chi-squared and Fischer exact test, when needed, and evaluated the association level between organ complication and clinical individual risk factors through univariate analysis. We included the risk factors for being hospitalized having Chagas disease and a form of organ affectation with a statistically significant association (p<0.1) in a multivariable logistic regression model (backward stepwise procedure), in order to control for possible confounders. Data analysis was performed using STATA software version 12.

### Ethics Statement

This study involves use of patient medical data from The Spanish National Hospital Database (CMBD). These data, are hosted by the Ministry of Health Social Services and Equality (MSSSI). Researchers working in public and private institutions can request the databases by filling, signing and sending a questionnaire available at the MSSSI website. In this questionnaire a signed Confidentiality Commitment is required. All data is anonymized and de-identified by the MSSII before it is provided to applicants. According to this Confidentiality Commitment signed with the MSSSI, researchers cannot provide the data to other researchers, thus other researchers must request the data directly to the MSSSI [22].

## Results

### Distribution of Hospitalization Related to Chagas by Time and Autonomous Region

Between 1997 and 2011, 1729 hospitalizations including Chagas disease in any diagnosis position were recorded in Spain, out of which 546 (31.6%) were re-admissions. Up to 90% (1630/1729) of these cases were notified between 2005 and 2011. For the two study periods (1998–2004 and 2005–2011), we obtained hospitalization rates of 18 and 242.8/100000 population, respectively.

**Fig 1:**
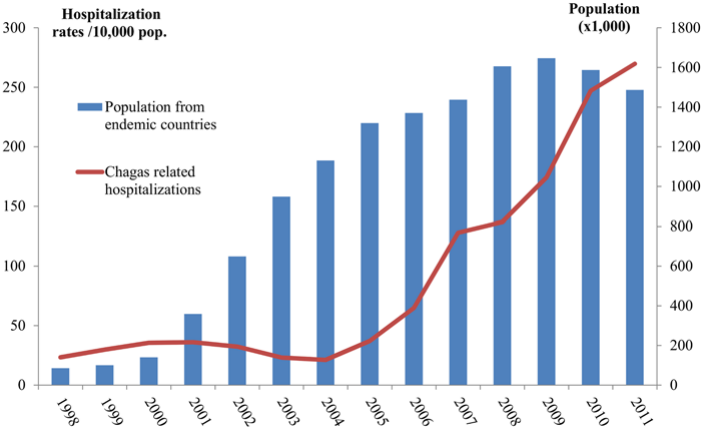
Immigrant population coming from Chagas disease endemic countries and Chagas related hospitalization rates 1998–2011, Spain.

**Fig 2:**
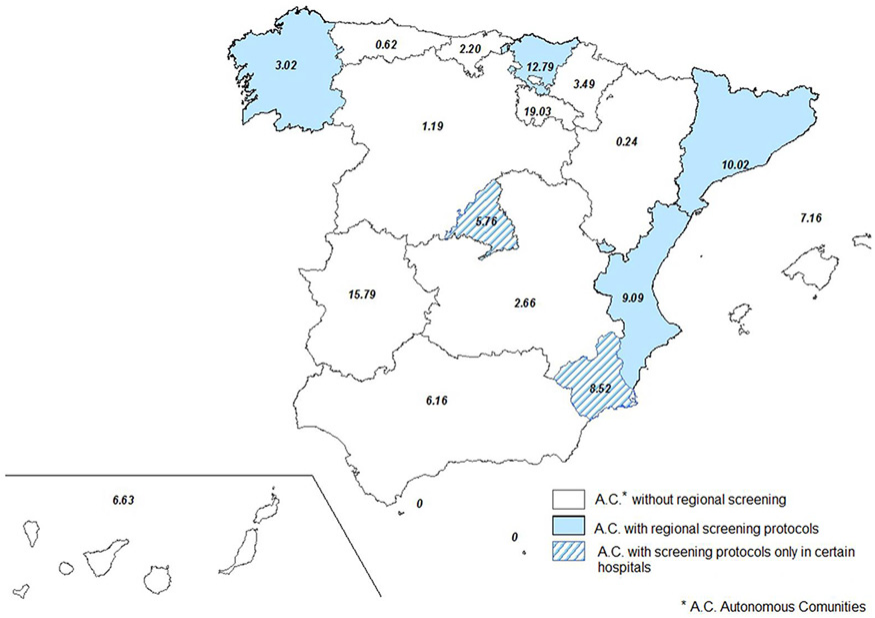
Increase in Chagas disease hospitalization rates by region between 1998–2004 and 2005–2011, Spain (times).

**Fig 3:**
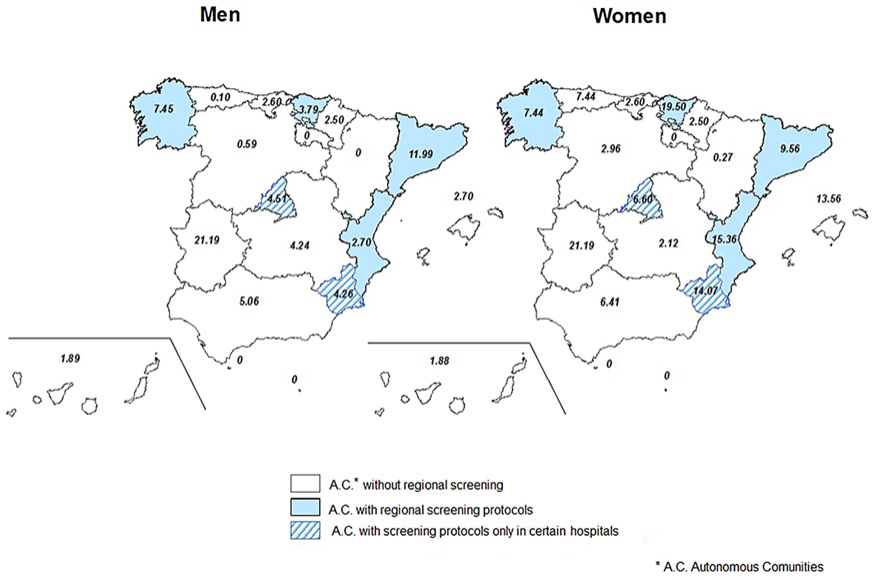
Increase in Chagas disease hospitalization rate by region and sex between 1998–2004 and 2005–2011 by sex, Spain (times).

While the Latin American population migrating form Chagas disease endemic countries began to increase steadily from 2001, the hospitalization rate showed a stationary tendency in the first time period. In the second time interval (2005–2011), this rate increased while the Latin American population began a slow decrease, starting with 2009 (Fig 1). The increase in hospitalization rate was registered in all but two autonomous regions (Fig 2 and S2 Table). For eight autonomous regions the hospitalization rate increased more for women, in two increased more for men, while in seven autonomous regions there was no difference among sexes (Fig 3).

### Clinical Characteristics of Chagas Related Hospitalizations

**Fig 4:**
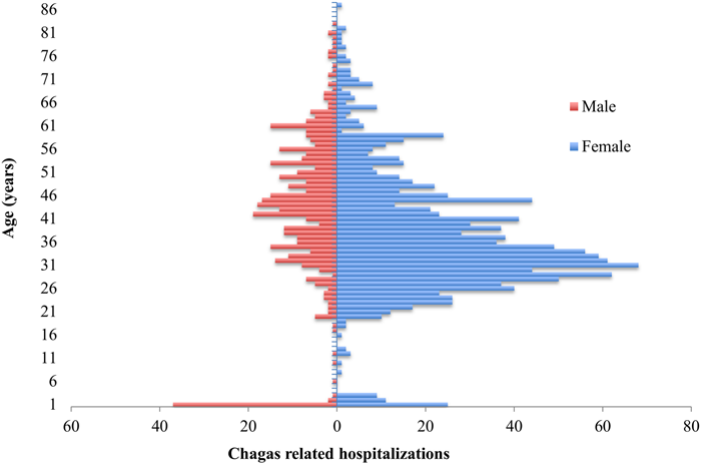
Distribution of Chagas disease related hospitalization by sex, Spain, 1998–2011.

The median age of the 1729 hospitalizations was 35 years (range 0–87) with the 16–45 age-group being mostly represented with 1207/1729 (69.8%). A total of 74% hospitalized were female, predominating in all age-groups (Fig 4). For 206/1729 (11.9%) records, Chagas disease was registered as the first diagnosis while for 878/1729 (50.7%) appeared as second diagnosis. Other diagnoses in first position are summarized in S1 Table. In the remaining 37.4% records, Chagas disease appeared in any of the last 12 positions. The most frequent main diagnostics associated with Chagas disease were related to pregnancy, giving birth or postpartum complications (36.6%), heart and circulatory conditions (15.3%) and digestive system conditions (9.1%). The hospital departments with higher number of Chagas related admissions were Obstetrics-Gynecology (37.1%), Cardiology (13.1%), Internal Medicine (11.2%) and Digestive Diseases (7.9%) (S3 Table). Overall, 1302/1792 (75.3%) hospitalizations were related to Chagas disease without organ complications, while 388/1792 (23.4%) hospitalizations were recorded as Chagas disease with organ complications, of which 317/388 (81.7%) were due to heart complications and 71/388 (17.9%) were due to another organ complications. Invasive or non-invasive medical procedures were documented for 1463/1729 (84.6%) hospitalizations. We identified 290 different types of medical procedures; 579/1463 (33.5%) procedures were related to obstetrical surgery, 375/1463 (21.7%) were diagnosis or therapy procedures and 190/1463 (11%) were related to heart surgery. Of the total 1729 hospitalizations, 487 (28.2%) had registered a surgical intervention. The admission period was inferior to one week in 71% of Chagas related hospitalizations. The predominant admission type was urgent (76.6%) and 61.5% had a severity level of 2 (out of 4). Around 95% of them were discharged at home, decease occurring in 1.2% of overall Chagas related hospitalizations. The hospitalization median cost was 3,064.9 euros (range 287.3–939,324.1). Costs were covered by social security health care service for 95% of the cases (Table 2).

**Table 2:** Clinical characteristics of all hospitalizations (n = 1729), hospitalizations including Chagas disease as first diagnosis (n = 206) and hospitalizations including child delivery as first diagnosis (n = 614), Spain 1997–2011.

	Hospitalizations including Chagas disease diagnostic					
Variables	Chagas disease at any diagnostic position (n = 1729)		Chagas disease as first diagnosis (n = 206)		Child delivery as first diagnosis (n = 614)	
	n	%	n	%	n	%
**Sex**						
Male	450	26.03	92	44.88	N.A.	N.A.
Female	1279	73.97	114	55.61	614	100
**Age**						
≤5 years	86	4.97	21	10.24	N.A.	N.A.
6–15 years	10	0.58	4	1.95	1	0.16
16–45 years	1207	69.81	101	49.27	612	99.67
46–65 years	363	20.99	67	32.68	1	0.16
≥66 years	63	3.64	13	6.34	N.A.	N.A.
**Chagas disease type**						
Without organ affectation	1302	75.30	77	37.56	587	95.60
With heart complication	317	17.29	89	43.41	23	3.75
With other organ complication	71	4.11	34	16.59	4	0.65
Heart and other organ complication	18	1.04	1	0.49	0	0
Unknown	39	2.26	5	1.95	0	0
**Surgical intervention history**						
No	1242	71.83	181	88.29	345	56.19
Yes	487	28.17	25	12.20	269	43.81
**Admission type**						
Urgent	1316	76.11	120	58.54	572	93.16
Programed	403	23.31	85	41.46	42	6.84
Unknown	10	0.58	1	0.49	0	0
**Discharge type**						
Home	1652	95.55	184	89.76	611	99.51
Other	56	3.24	17	8.29	3	0.49
Deceased	21	1.21	5	2.44	0	0
**Re-admission**						
No	1183	68.42	118	57.07	584	95.11
Yes	546	31.58	88	42.93	30	4.89
**Hospitalization time**						
<one week	1233	71.32	114	55.6	585	95.28
>one week	457	26.43	91	45.4	29	4.72
**Costs covered by social security health care**						
No	87	5.03	15	7.28	27	4.40
Yes	1642	94.97	191	92.72	609	95.60
**Variable**	**Median**	**Range**	**Median**	**Range**	**Median**	**Range**
Hospitalization time (days)	4	0–231	7	0–231	3	1–27
Hospitalization cost (euro)	3,064.9	939,036.78	4,195.62	41,8549.38	2,207.86	376,733.68

### Chagas Disease as First Diagnosis

A total of 55.6% (114/206) hospitalized were female, with the 16–45 and 46–65 age-groups being mostly represented (49.3% and 32.7%, respectively). Around 50% of the hospitalizations were related to Chagas disease with organ complication. Surgical intervention was documented for 25/206 (12.2%) hospitalizations and invasive or non-invasive medical procedures were documented for 147/206 (71.4%) hospitalizations. The predominant admission type was urgent (58.5%) followed by programmed (41.5%). Median time of hospitalization was 7 days, and up to 40% stayed more than one week. Around 90% (184/206) were discharged at home; decease occurred in 2.4% of these hospitalizations. The hospitalization median cost was 4195.6 euros.

### Child Delivery as First Diagnosis

From 1997 to 2011, 614 hospitalizations related to Chagas disease with child delivery as first diagnosis occurred. The 16–45 age-group was predominant with 612/614 (99.7%) and heart and other organ complications were present in 23/614 (3.8%) and 4/614 (0.6%) of these hospitalizations, respectively. Surgical intervention was recorded for 43.8% of them, and they were mostly urgent admissions (93.2%). Invasive or non-invasive medical procedures were documented for all the hospitalizations, mainly obstetrical procedures. Up to 95% stayed less than one week in the hospital, being the median average time of 3 days. Around 99% hospitalizations were discharged at home, only 30/614 (4.9%) need to be re-admitted. The hospitalization median cost was 2,207.9 euros.

### Conditions Associated with Chagas Disease with Organ Complications

Through bivariate analysis, we identified several conditions associated with hospitalization due to Chagas disease with organ affectation. Being male, aged over 45 years, Chagas disease as main diagnosis, a history of re-admission in the hospital, and hospitalization’s cost higher than the median (3,065 euros), were significantly associated with Chagas disease with organ affectation. On the other hand, being aged between 16 and 45 years, hospitalization stay up to one week, a history surgery, the urgent admission type and the medium level of disease severity where inversely associated with organ affectation (Table 3).

**Table 3:** Factors associated with organ complication in hospitalizations including Chagas disease, Spain, 1997–2011.

Variables		Organ complication (n = 388)		Without organ complication (n = 1,302)		unadjusted OR	p value	adjusted OR	p value
		n	%	n	%				
Sex	Male	160	41.20	278	21.30	1	0.000	1	0.048
	Female	228	58.80	1,024	78.70	2.58 (2.02–3.29)	0.000	1.33 (1.00–1.77)	0.048
Age group	≤5	23	5.93	58	4.45	1		1	1
	6–15	0	0.00	10	0.77	−	−	−	−
	16–45	177	45.62	1,003	77.04	0.44 (0.27–0.74)	0.002	0.97 (0.54–1.72)	0.916
	46–65	159	40.98	199	15.28	2.01 (1.19–3.41)	0.009	2.59 (1.42–4.71)	0.002
	≥66	29	7.47	32	2.46	2.29 (1.14–4.59)	0.020	2.57 (1.19–5.54)	0.010
Chagas as first diagnosis	Yes	128	33.00	77	5.90	1	0.000	1	0.000
	No	260	67.00	1,225	94.10	7.83 (5.72–10.7)	0.000	5.42 (3.73–7.86)	0.000
Hospitalization time	≤1 week	224	57.70	1,009	77.50	1	0.000	1	N.S.
	>1 week	164	42.30	293	22.50	0.40 (0.31–0.50)	0.000	N.S.	N.S.
Surgical intervention	Yes	59	15.20	417	32.00	1	0.000	1	0.003
	No	329	84.80	885	68.00	0.38 (0.28–0.51)	0.000	0.58 (0.41–0.83)	0.003
Re-admission	Yes	51	13.10	106	8.10	1	0.000	1	0.000
	No	337	86.90	1,196	91.90	4.00 (3.16–5.07)	0.000	2.86 (2.17–3.78)	0.000
Admission type	Urgent	263	68.10	1022	79.00	1	0.000	1	N.S.
	Planned	123	31.90	272	21.00	0.57 (0.44–0.73)	0.000	N.S.	N.S.
Deceased	Non-exitus	380	97.94	1289	99.00	1	0.101	1	N.S.
	Exitus	8	2.06	13	1.00	2.10 (0.87–5.12)	0.101	N.S.	N.S.
Hospitalization cost	≤3,064.9	91	23.50	745	57.20	1	0.000	1	0.000
	>3,064.9	297	76.50	557	42.80	4.24 (3.28–5.48)	0.000	2.03 (1.49–2.78)	0.000
Severity level	0–1	69	17.8	111	8.50	1		1	
	2	173	44.6	871	66.90	0.32 (0.23–0.45)	0.000	0.46 (0.3–0.7)	0.001
	3–4	146	37.6	320	24.60	0.73 (0.51–1.05)	0.091	0.73 (0.46–1.15)	0.176

N.S.- Non significant; Goodness of fit: AIC = 1389.17; Pseudo R^2^ = 0.24;

All these significant variables where included in the final multivariable logistic regression model. Results indicated that the patients being hospitalized with organ affectation Chagas disease were 1.3 times more likely to be male, 2.6 times more likely to be aged between 45 and 64 years, 5.4 times more likely to be admitted with Chagas disease as main diagnosis, and almost 3 times more likely to be admitted for the first time in the hospital, compared to those admitted with Chagas disease without organ affectation. Also, hospitalizations with organ affectation were two times more likely to cost above the median cost of 3065 euro that the hospitalizations of asymptomatic Chagas disease. Not having a personal surgery history and medium severity level appeared to have a negative association with Chagas disease with organ affectation (Table 3).

## Discussion

Although there are few studies performed locally in Spain [16,26,27], to our knowledge, this is the first one addressing Chagas disease epidemiology nationwide. Moreover, the use of hospitalized data for characterizing Chagas disease epidemiology in non-endemic countries also represents a premier. We relied on a database providing information from a net of hospitals that covers more than 98% of the population living in Spain [24], therefore we belief that our results are representative for the Spanish territory.

We aimed to describe the Chagas disease epidemiology between 1998 and 2011 in Spain. As CMBD do not provide enough information that would allow for differentiation between *T*. *cruzi* infection and Chagas disease, we have chosen the second one as term for our discussion. We have observed an overall increase in hospitalizations including Chagas disease, beginning with 2005 (Fig 3). This occurs while the figures for Latin American migrant population from countries endemic to Chagas were increasing in the 2000–2004 period, but become stable after 2005. This increase in hospitalizations that includes Chagas disease might be due to the introduction in 2005 of the national program for controlling the Chagas disease horizontal transmission (blood and organ donors screening). Another explanation might surge from the implementation of screening programs for pregnant women. However, it is difficult to quantify the impact of these measures over the hospitalization rate. Community of Valencia in 2007 [18], Catalonia in 2010 [19,28], Galicia in 2012 [20], Basque Country in 2008 [21] and more recently Murcia in 2013 [29] (5 out of 17) have implemented protocols for screening and diagnosis of *T*. *cruzi* infection in pregnant women in order to allow for early diagnostic and infection treatment in newborns. The hospitalization rates increased more for women in Basque country, Murcia and Valencian Community, while the increase′s rates were higher in men in Catalonia and similar for both sexes in Galicia. In the Basque Country the recommendation for screening pregnant women from endemic countries includes only those women with clinical symptoms or EKG modifications [21]. Protocols also exist in other autonomous communities, but their implementation is confined only to certain hospitals. This is the case of Madrid [30] and Murcia (before the implementation of the regional screening in 2013) [31]. These protocols are heterogeneous in terms of target population as well as the region coverage and the year of implementation (Table 1).

Otherwise, the number of hospitalizations has also increased in those communities without official screening practices and/or regulations in place for pregnant women. Increased awareness of Chagas disease in both healthcare professionals and patients and relatives might be a possible explanation for this trend. It is also feasible that local initiatives towards early diagnosis of Chagas disease might exist in other autonomous regions, these actions remaining unknown at central level. It remains however unclear why in two communities the overall hospitalization rate decreased in 2005–2011 compared to 1998–2004.

Our study indicates that most of the hospitalizations which included Chagas disease diagnosis at any position were females. Nevertheless, the percentage of female sex decreased from 74% to 55.6% when we analyzed only those hospitalizations with Chagas disease as first diagnosis. Another striking feature is the age-group, as most of the female cases were aged between 16–45 years, while the immigrant population distribution in Spain, reveals however uniformity between sexes [32]. Similar findings were described in another Spanish study on patients with Chagas disease attending the hospital care [33]. This can have several explanations: according to the Spanish Institute of Statistics, the migrant Latin American women are predominant in Spain, the 16–45 age-group corresponding to the most well represented age group, with more than 70% being aged between 15 and 45 years [25]. Additionally, screening programs targeting pregnant women implemented in the above mentioned autonomous communities could represent an alternative explanation for the high percentage of hospitalized women at fertile age. This is concordant with a previous estimation indicating that women at childbearing age represent more than 60% of the overall estimated *T*. *cruzi* infections in Spain [10]. This finding supports even more the need of extended screening for pregnant women coming from Latin American countries endemic for Chagas. On the other hand, when we assessed only those hospitalizations with Chagas disease as first diagnosis (n = 206), the 16–45 years old group remain the most frequent (49.3%), but closely followed by the 46–65 years old (32.7%). These figures are similar with the Chagas’ disease prevalence rates provided by WHO Global burden of Chagas’ disease estimations [34].

The most important aspect in Chagas disease evolution is represented by the heart or digestive system affectation, secondary to prolonged *T*. *cruzi* infection. In our study, the percentage of the reports with cardiac or digestive affectation was around 22%. Of these hospitalizations, the majority had developed a Chagas related cardiac chronic condition. These figures are concordant with a previous estimate of potentially infected immigrants in Spain [10,35]. On the other hand, the high percentage of diagnosed asymptomatic *T*.*cruzi* in our study (77%) could be seen as due to implementation of nationwide and local screening programs. These is however difficult to prove. The analyzed database do not registers information related to the reason of testing for *T*. *cruzi* existence. Moreover, the rates of symptomatic Chagas disease increase to 50% when we assess only those hospitalizations with Chagas disease as first diagnosis, the heart being the main affected organ. This supports our hypothesis and also bring to light that Chagas disease could be consider a reason for hospitalization, even if organic complications do not exist. A possible explanation might be that these hospitalizations are related with diagnostic procedures. In fact, 74% of these hospitalizations include invasive or non-invasive medical procedures.

The majority of hospitalizations (1524/1729) didn’t have Chagas disease recorded as the main diagnosis; therefore one could consider that even though Chagas disease was present as pathology, it was not the main reason for hospital admission. Almost half of the hospitalizations with Chagas disease as main principal diagnosis had documented chagasic heart condition and another 17% other organ affectation. On the other hand, for the reports where Chagas is not the main diagnosis, 82% had documented asymptomatic Chagas disease. Moreover, less than 5% of the hospitalizations with child delivery as first diagnosis include Chagas disease with any organ complication. Therefore one could speculate that unless the *T*. *cruzi* has evolved into a chronic condition, it represents most of the time a silent disease and can easily go under-diagnosed if not properly searched for. This again stresses the importance of developing efficient strategies for an early *T*.*cruzi* detection, even more when considering that the efficacy of Chagas disease drugs diminishes the longer a person has been infected [4].

History of surgical intervention was especially prevalent in child delivery hospitalizations, probably related to the delivery itself, as it occurred with the admission type, most commonly urgent in this kind of hospitalizations. On the other hand, hospitalizations with Chagas disease as first diagnosis stayed longer in the hospital than those where it was placed at any position or where child delivery was listed at first diagnosis. Moreover, we saw that the percentage of deaths was slightly higher in those reports with Chagas disease as first diagnosis. We know that Chagas disease is a major cause of mortality in Latin America due to a wide range of pathogenic processes [36]. Our results might suggest higher severity (and probably more symptomatic) if Chagas is placed as first diagnostic, however this is difficult to prove. The analyzed database do not registers information related to the cause of death. Finally, the median cost was 37% and 90% more in hospitalizations with Chagas disease as first diagnosis compared to records with Chagas diseases in any diagnostic position and delivery as first diagnosis, respectively. This finding suggests that the expansion of the screening program and other prevention activities might be a good public health policy in concept of saving costs.

We have identified the male gender as being associated with Chagas disease involving organ affectation. The association was borderline significant, indicating a possible similarity between genders when it comes to chronic Chagas disease. Reports on sex differences in progressive Chagas’ disease are controversial and previous studies have found either that it is more common in men or that it is unrelated to sex [37]. Eventually, selective preventive measure such as Chagas screening only and exclusively in pregnant women might also result in gender differences.

The association of the 45–64 years age-group with Chagas disease with organ affectation is concordant with previous knowledge related to a 20–30 years of asymptomatic infection between contracting the infection and developing the chronic form [38]. Also reports relating Chagas disease with organ conditions were associated with having Chagas disease as main diagnosis and being hospitalized for the first time. Chagas disease associated to an organ condition was 2 times more likely to have a hospitalization cost above the median value than those without organ complications, this finding being especially relevant from a public health perspective for Spain. The cost could be even higher if one is considering the indirect costs related to work absenteeism, incapacity, etc. This might be also the case in any non-endemic country with important immigration from Latin America. Sicuri *et al*. indicated in their economic evaluation of Chagas disease screening in Catalonia, that targeting all Latin American women giving birth in Spain and of their infants is the best strategy compared to the non-screening option, showing not only obvious economic advantages but also providing useful information for health policy makers in their decision making process [39].

### Limitations and Conclusions

We calculated the hospitalization rate using as denominators Latin American immigrants officially registered in Spain. Non-registered immigrants could enlarge both the population denominator and the number of cases, thus the results should be interpreted with caution. Another limitation when using the CMBD database is that the country of origin is not recorded, therefore the hospitalization rates can be calculated only as overall figures. This is an important aspect being known that the endemicity levels varies widely among Latin American countries [40]. On the other hand, the estimation of the risk factors for hospitalizations with organ complications might be influenced by this and other factors not present in the CMBD database.

One of the strong points of CMBD is that it provides the total number of hospitalizations records without being subject to the limitations of outpatient surveillance systems, such as under-diagnosis or reporting deficiencies. It remains dependent only by the population’s health seeking behavior and healthcare accessibility. The Spanish Government enacted a Royal Decree in 2012 which basically limits access to free services at the point of delivery for all population (including non-residents migrants), undermining the principle of universal coverage which exist in this country since 1986 [41]. This can have as consequence a decrease in the number of Latin American immigrants attending to the healthcare services. Subsequently, and in the absence of systematic and coordinated actions towards early detection of *T*. *cruzi*, a larger number of asymptomatic infections might remain under-diagnosed, increasing the number of patients suffering from Chagas disease chronic conditions in the future.

In conclusion, using hospitalized cases discharge database can be a useful tool when describing the Chagas disease epidemiology. However, our work underlines the need of a nationwide systematic approach towards *T*. *cruzi* early detection, especially targeting fertile aged women and relatives from endemic Latin American countries. Health inequalities on immigrants health status might exist across our country if screening programs differ from one region to another and, furthermore, are not nationwide implemented. Moreover, a national surveillance system that would allow a more accurate data collection, analyzing and interpretation, could be of added value in completing a more accurate picture of Chagas disease in Spain, resulting useful both in gaining extended disease knowledge, but especially in evaluating implemented control actions.

## Supporting Information

S1 Checklist STROBE checklist. (DOC)

S1 Table Diagnosis in first position in all hospitalizations records including Chagas disease in Spain from 1997 to 2011. (DOCX)

S2 Table Chagas related hospitalization rates per 100.000 population at risk by region in two time periods, Spain. (DOCX)

S3 Table Hospital departments with higher number of Chagas related admissions, 1997–2011, Spain. (DOCX)
