# Supplementary material for: Using Hospital Discharge Database to Characterize Chagas Disease Evolution in Spain: There Is a Need for a Systematic Approach towards Disease Detection and Control
Source: PLoS Negl Trop Dis. 2015 Apr 17;9(4):e0003710. doi: 10.1371/journal.pntd.0003710 (PMC4401715; doi:10.1371/journal.pntd.0003710)
Supplement: S1 Table — (DOCX) [file pntd.0003710.s002.docx]

| **Diagnosis in first position*** | **ICD-9** | **n** | **%** |
| --- | --- | --- | --- |
| Chagas disease with heart complication | 086.0 | 91 | 5.3 |
| Chagas disease with other organ complication | 086.1 | 36 | 2.1 |
| Chagas disease without organ affectation | 086.2 | 78 | 4.5 |
| Undetermined chagas disease | 0.86.9 | 40 | 2.3 |
| Complete atrioventricular block | 426.0 | 24 | 1.4 |
| Paroxysmal ventricular tachycardia | 427.1 | 23 | 1.3 |
| Sinoatrial node dysfunction | 427.81 | 21 | 1.2 |
| Unspecified congestive heart failure | 428.0 | 41 | 2.4 |
| Gallbladder stones (not mentioning cholecystitis) | 574.20 | 33 | 1.9 |
| Embarazo postérmino | 645.11 | 57 | 3.3 |
| Other specified infectious and parasitic diseases complicating pregnancy, childbirth or puerperium | 647.81 | 119 | 6.9 |
| Previous cesarean section | 654.21 | 29 | 1.7 |
| Other specified fetal and placental problems | 656.81 | 24 | 1.4 |
| Premature rupture of membranes | 658.11 | 64 | 3.7 |
| Prolonged second stage of labor | 662.21 | 17 | 1 |
| ^[[1]](#footnote-1)^Others | --- | 1.062 | 61.9 |
| **Total** |  | **1729** | **100** |
| *Only those diagnoses that appear in at least 1% of hospitalizations are shown. | | | |

**Supplementary table 1. Diagnosis in first position in all hospitalizations records including Chagas disease**

1. [↑](#footnote-ref-1)
