# Supplementary material for: Using Hospital Discharge Database to Characterize Chagas Disease Evolution in Spain: There Is a Need for a Systematic Approach towards Disease Detection and Control
Source: PLoS Negl Trop Dis. 2015 Apr 17;9(4):e0003710. doi: 10.1371/journal.pntd.0003710 (PMC4401715; doi:10.1371/journal.pntd.0003710)
Supplement: S2 Table — (DOCX) [file pntd.0003710.s003.docx]

| **Supplementary Table 2. Chagas related hospitalization rates per 100.000 population at risk by region in two time periods, Spain** | | | |
| --- | --- | --- | --- |
| **Autonomous region** | **First period (1998-2004)** | **Second period (2005-2011)** | **Hospitalization rates increase (times)** |
|  |  |  |  |
| Andalusia | 16.4 | 278.1 | 17 |
| Aragon | 81.3 | 44.3 | 0.5 |
| Asturias | 61 | 90.7 | 1.5 |
| Balearic Islands | 11.3 | 183.5 | 16.2 |
| Canary Islands | 3.9 | 49.4 | 12.7 |
| Cantabria | 0 | 41.4 | 41.4 |
| Castilla y León | 75 | 228.6 | 3 |
| Castilla-La Mancha | 30.5 | 208.1 | 6.8 |
| Catalonia | 17.3 | 426.7 | 24.7 |
| Extremadura | 0 | 281.2 | 281.2 |
| Galicia | 14.4 | 94.1 | 6.5 |
| Madrid | 13.9 | 125.6 | 9 |
| Murcia | 18 | 320.1 | 17.8 |
| Navarra | 0 | 45.8 | 45.8 |
| La Rioja | 0 | 297.4 | 297.4 |
| Basque Country | 9.2 | 370 | 40.2 |
| Valencian Community | 20.7 | 413.9 | 20 |
| Ceuta & Melilla | 0 | 0 | 0 |
| **Total** | **18.0** | **242.8** | **13.5** |
