# Supplementary material for: Using Hospital Discharge Database to Characterize Chagas Disease Evolution in Spain: There Is a Need for a Systematic Approach towards Disease Detection and Control
Source: PLoS Negl Trop Dis. 2015 Apr 17;9(4):e0003710. doi: 10.1371/journal.pntd.0003710 (PMC4401715; doi:10.1371/journal.pntd.0003710)
Supplement: S3 Table — (DOCX) [file pntd.0003710.s004.docx]

Supplementary table 3. Hospital departments with higher number of Chagas related admissions, 1997-2011, Spain.

| **Main Hospital Service** | **Chagas related admissions (n=1729)** | |
| --- | --- | --- |
|  | **n** | **%** |
| **Gynecology** | 57 | 3.30 |
| **Obstetrics** | 585 | 33.83 |
| **Cardiology** | 226 | 13.07 |
| **Internal Medicine** | 204 | 11.80 |
| **Digestive** | 60 | 3,47 |
| **Digestive surgery** | 77 | 4.45 |
| **Pediatrics** | 60 | 3.47 |
| **Unregistered** | 115 | 6.65 |
| **Others departments** | 345 | 19.95 |
